# FUT1 deficiency elicits immune dysregulation and corneal opacity in steady state and under stress

**DOI:** 10.1038/s41419-020-2489-x

**Published:** 2020-04-24

**Authors:** Kyoung Woo Kim, Jin Suk Ryu, Jung Hwa Ko, Jun Yeob Kim, Hyeon Ji Kim, Hyun Ju Lee, Jang-Hee Oh, Jin Ho Chung, Joo Youn Oh

**Affiliations:** 1 Department of Ophthalmology, Chung-Ang University Hospital, Seoul, South Korea; 2 Laboratory of Ocular Regenerative Medicine and Immunology, Seoul Artificial Eye Center, Seoul National University Hospital Biomedical Research Institute, Seoul, South Korea; 3 Department of Dermatology, Seoul National University College of Medicine, Seoul, South Korea; 4 Department of Ophthalmology, Seoul National University College of Medicine, Seoul, South Korea

**Keywords:** Inflammatory diseases, Inflammatory diseases, Inflammation, Inflammation

## Abstract

Fucosylation is a biological process that plays a critical role in multiple cellular functions from cell adhesion to immune regulation. Fucosyltransferases (FUTs) mediate fucosylation, and dysregulation of genes encoding FUTs is associated with various diseases. FUT1 and its fucosylated products are expressed in the ocular surface and ocular adnexa; however, the role of FUT1 in the ocular surface health and disease is yet unclear. Here, we investigated the effects of FUT1 on the ocular surface in steady-state conditions with age and under desiccating stress using a *Fut1* knockout (KO) mouse model. We found that corneal epithelial defects and stromal opacity developed in *Fut1* KO mice. Also, inflammatory responses in the ocular surface and Th1 cell activation in ocular draining lymph nodes (DLNs) were upregulated. Desiccating stress further aggravated Th1 cell-mediated immune responses in DLNs, lacrimal gland, and ocular surface in *Fut1* KO mice, leading to severe corneal epithelial disruption and opacity. Mixed lymphocyte reaction assays revealed that the activity of splenocytes to stimulate CD4 T-cell proliferation was increased in *Fut1* KO mice. Together, these data demonstrate that FUT1 deficiency induces immune dysregulation in the ocular surface and corneal opacity in steady state and under desiccating stress.

## Introduction

Glycans are involved in a wide variety of physiologic and pathologic processes in eukaryotic cells after attachment to proteins or lipids through an enzymatic process called glycosylation^1^. In particular, fucosylated carbohydrates play an important role in the regulation of multiple cellular functions such as cell trafficking, immune cell development, and interaction with gut microbes and are generated through fucosylation mediated by fucosyltransferases (FUTs)^2–4^. Thus far, 13 FUT genes have been identified in human genome based on their acceptor specificities and protein sequences^5^. Among them, *Fut1* and *Fut2* genes encode galactoside 2-alpha-L-fucosyltransferase that mediates the addition of L-fucose to the terminal β-D-galactose residues of glycan via α1,2 linkage. While *Fut2* gene is expressed in limited tissues^6^, *Fut1* gene exhibits more broad expression in about 37 types of human tissues including stomach, lung, and pancreas according to the data from genome sequencing^7^ and HPA RNA-seq (https://www.proteinatlas.org).

Previous studies have demonstrated that FUT1 mediates diverse biologic processes by inducing angiogenesis and macrophage polarization in rheumatoid arthritis^8–10^, promoting keratinocyte migration^11^, and increasing cancer cell survival in breast and liver cancers^12,13^. In the ocular surface, several types of glycoproteins have been reported to be expressed on the corneal epithelium of rats, rabbits, and humans^14–16^. Also, a previous study showed that topical application of fucose accelerated corneal epithelial wound healing in a rabbit model of iodine vapor-induced corneal burn^17^. Yet the role of FUT1-mediated fucosylation in the ocular surface has not been elucidated. Herein, we capitalized upon a *Fut1* knockout (KO) mouse model and investigated the effects of FUT1 and its product H antigen on the ocular surface in age-dependent steady-state conditions and under desiccating stress.

## Results

### *Fut1, Fut2*, and H2 antigen are expressed in the ocular surface and ocular adnexal tissues

**Fig. 1:**
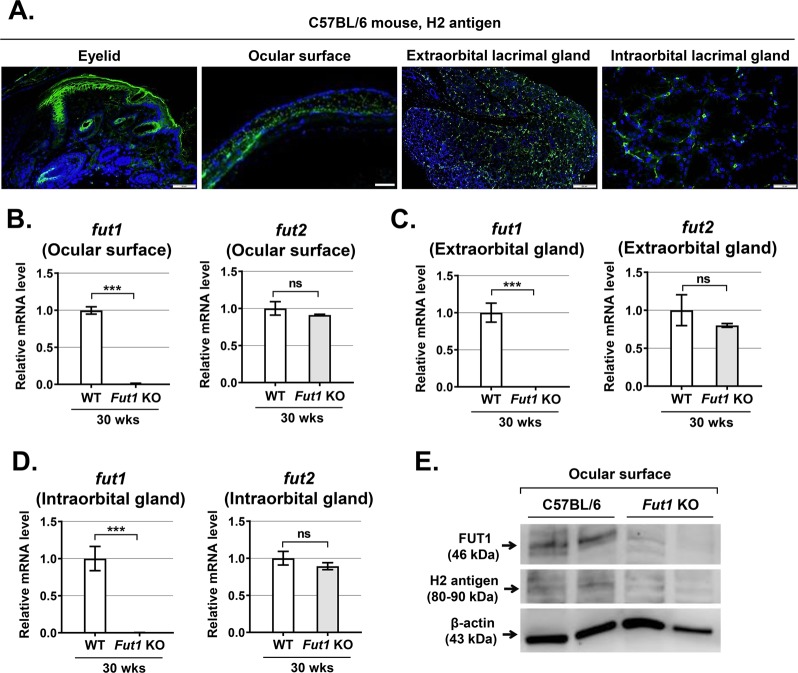
Expression of H2 antigen and FUT1 in the ocular surface, eyelid, and lacrimal glands in mice. **a** Immunostaining for H2 antigen in the eyelid, ocular surface, extraorbital and intraorbital lacrimal glands in C57BL/6 mice. **b–d** Real-time RT-PCR analysis for *Fut1* and *2* genes in the ocular surface, extraorbital and intraorbital lacrimal glands of *Fut1* knockout (KO) mice vs wild-type (WT) C57BL/6 mice. Shown are the relative mRNA levels of each gene in *Fut1* KO mice to those in WT mice (mean ± SEM). **e** The representative western blot image for FUT1 protein and H2 antigen in the ocular surface (cornea and conjunctiva) of *Fut1* KO vs WT mice. ***p < 0.001, ns: not significant. Student’s t-test was performed for statistical analysis.

We first evaluated the expression of *Fut1, Fut*2, and H2 antigen in the ocular surface and ocular adnexal tissues in C57BL/6 mice (Fig. 1). H2 antigen, which is synthesized by α1,2-fucosyltransferase encoded by *Fut1* and *Fut*2 genes^18^, was expressed in the ocular surface, eyelid, extraorbital lacrimal gland, and intraorbital lacrimal gland as assessed by immunohistochemical staining (Fig. 1a). Also, both *Fut1* and *Fut*2 genes were expressed in the ocular surface (containing the cornea and conjunctiva), extraorbital lacrimal gland, and intraorbital lacrimal gland as examined by real-time RT-PCR (Fig. 1b–d).

Next, we analyzed the expression of *Fut1, Fut*2, and H2 antigen in *Fut1* KO mice (B6.129-*Fut1*^tm1Sdo^/J) in comparison to wild-type (WT) C57BL/6 control mice. As expected, *Fut1* gene expression was abolished in the ocular and adnexal tissues in *Fut1* KO mice as measured by real-time RT-PCR, while *Fut2* gene was unaltered (Fig. 1b–d). Similarly, the protein expression of FUT1 and H2 antigen detected by western blot was markedly decreased in the ocular surface (cornea and conjunctiva) in *Fut1* KO mice, compared with WT mice (Fig. 1e).

### FUT1 deficiency induces corneal epithelial disruption and stromal opacity in steady state

**Fig. 2:**
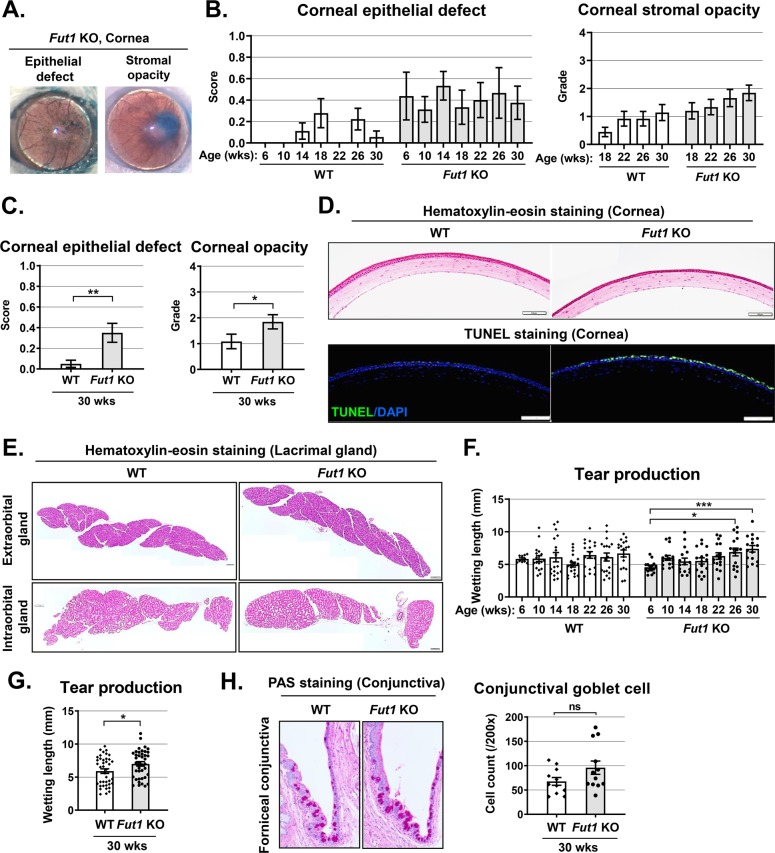
Longitudinal observation and comparison of the ocular surface phenotype in *Fut1* KO vs WT C57BL/6 mice. **a** Representative corneal photographs of *Fut1* KO mice showing corneal epithelial defects and stromal opacity after lissamine green vital dye staining. The severity of corneal epithelial defects and opacity as graded by the standardized scoring systems were tracked with age from 6 to 30 weeks (wks) (**b**) and compared between *Fut1* KO and WT mice at 30 wks of age (**c**). **d** Representative hematoxylin-eosin and TUNEL staining images of the cornea in *Fut1* KO and WT mice at 30 wks of age. Scale bars: 100 μm. **e** Representative hematoxylin-eosin staining image of extraorbital and intraorbital lacrimal glands in *Fut1* KO and WT mice at 30 wks of age. Scale bar: 200 μm. **f**, **g** Amount of aqueous tear production as measured by a phenol red thread test in *Fut1* KO and WT mice with age. **h** Representative Periodic acid Schiff (PAS) staining images of the forniceal conjunctiva and the relevant conjunctival goblet cell counts in *Fut1* KO and WT mice. A dot depicts data from an individual mouse. Data are presented as mean ± SEM. *p < 0.05, **p < 0.01, ***p < 0.001, ns not significant. Mann–Whitney *U* test (**c**, **g**), Student’s *t*-test (**h**), and one-way ANOVA followed by Tukey’s *post-hoc* analysis (**f**) were performed for statistical analysis.

To investigate the role of FUT1 in the ocular surface, we made a serial observation of the corneal epithelial integrity, corneal stromal transparency, and aqueous tear production in *Fut1* KO mice every two weeks from 6 weeks until 30 weeks of age and compared with WT C57BL/6 mice (Fig. 2). Corneal epithelial defects were present in *Fut1* KO mice as observed after lissamine green vital staining while the corneal epithelium remained intact in WT mice during follow-up (Fig. 2a–c). Moreover, corneal stromal opacity developed more prominently in *Fut1* KO mice, compared with WT controls (Fig. 2a–c, Supplementary Table 1). Furthermore, hematoxylin-eosin and TUNEL (terminal deoxynucleotidyl transferase dUTP nick end labeling) staining of the cornea revealed that corneal epithelial thinning and apoptosis were more marked in *Fut1* KO mice (Fig. 2d).

To test whether corneal epithelial defects and stromal opacity observed in *Fut1* KO mice might be related to lacrimal gland dysfunction, we assayed for lacrimal gland histology and tear production. Hematoxylin-eosin staining showed that the structure of both extraorbital and intraorbital lacrimal glands was normal in *Fut1* KO mice (Fig. 2e), and aqueous tear secretion was not reduced but rather increased in *Fut1* KO mice, compared with WT controls as measured by a phenol red thread test (Fig. 2f, g). In addition, Periodic Acid Schiff (PAS) staining of the conjunctiva showed that there was no significant difference in the number of mucin-secreting goblet cells between *Fut1* KO and WT mice (Fig. 2h).

Together, the results demonstrate that FUT1 deficiency disrupts corneal epithelial integrity and induces corneal opacification without affecting lacrimal glands or conjunctival goblet cells in steady-state conditions.

### FUT1 deficiency upregulates ocular surface inflammation and regional Th1 cell activation

**Fig. 3:**
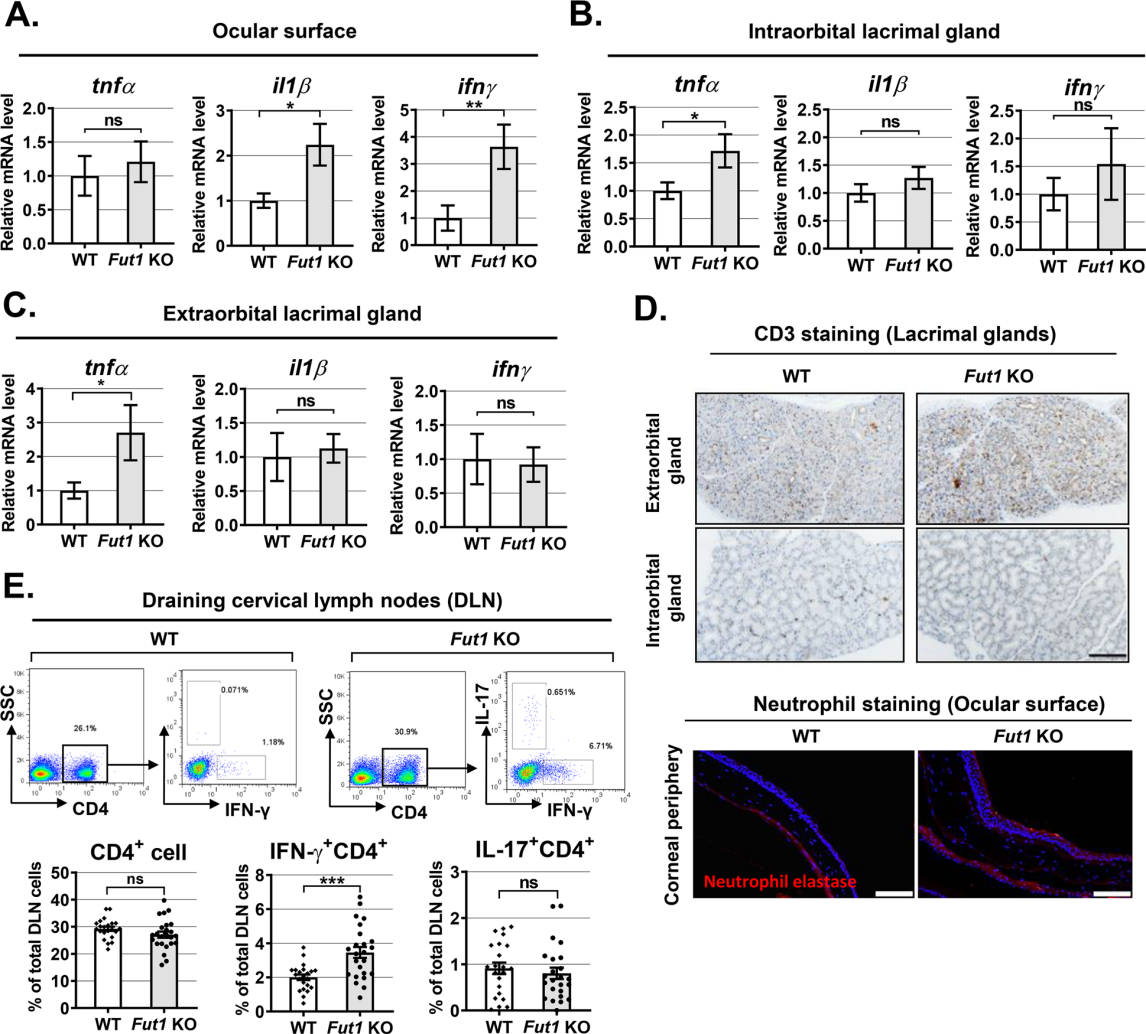
Inflammatory response in the ocular surface, lacrimal glands, and ocular draining cervical lymph nodes (DLN) in *Fut1* KO vs WT C57BL/6 mice. **a–c** Real-time RT-PCR analysis for mRNA levels of TNF-α, IL-1β, and IFN-γ in the ocular surface (cornea and conjunctiva), extraorbital and intraorbital lacrimal glands. Data are shown as the relative values of mRNA levels in *Fut1* KO mice to those in WT mice. **d** CD3 immunostaining of extraorbital and intraorbital lacrimal glands (Scale bar, 200 μm) and neutrophil staining of the cornea (Scale bar, 100 μm). **e** Representative and quantitative flow cytometry results for IFN-γ^+^CD4^+^ cells and IL-17^+^CD4^+^ cells in DLN. The percentage of CD4^+^, IFN-γ^+^CD4^+^ or IL-17^+^CD4^+^ cells out of total DLN cells is shown, and a dot depicts data from an individual mouse. Data are presented as mean ± SEM. *p < 0.05, **p < 0.01, ***p < 0.001, ns: not significant. Student’s *t*-test (IL-1β in **a**, **c**, **e**) and Mann–Whitney U test (TNF-α in A, and IFN-γ in B) were performed for statistical analysis.

The ocular surface integrity is closely related to inflammation. Thus, we next examined inflammatory responses in the ocular surface and lacrimal glands in 30-week-old *Fut1* KO and WT mice (Fig. 3). Real-time RT-PCR showed that mRNA levels of IL-1β and IFN-γ in the ocular surface containing the conjunctiva and cornea were significantly higher in *Fut1* KO mice than in WT mice (Fig. 3a). TNF-α level was significantly elevated in extraorbital and intraorbital lacrimal glands of *Fut1* KO mice compared with WT mice (Fig. 3b, c), but no differences were observed in IL-1β and IFN-γ mRNA levels and CD3^+^ T-cell infiltration in the glands between *Fut1* KO and WT mice (Fig. 3b–d). A few neutrophils were observed in the corneal epithelium in *Fut1* KO mice, whereas there was no neutrophil in the cornea in WT mice (Fig. 3d).

It is well-known that CD4^+^ T cells compromising the ocular surface integrity and driving lacrimal gland inflammation are activated in draining cervical lymph nodes (DLNs) and recruited to the ocular surface and glands^19^. We therefore analyzed DLNs for IFN-γ^+^CD4^+^ Th1 and IL-17^+^CD4^+^ Th17 cells by flow cytometry. Remarkably, the percentage of IFN-γ^+^CD4^+^ cells out of total DLN cells was significantly higher in *Fut1* KO mice than in WT controls, while the percentage of CD4^+^ or IL-17^+^CD4^+^ cells out of total DLN cells was not changed (Fig. 3e).

Hence, data indicate that FUT1 deficiency induces inflammation in the ocular surface and activates the regional Th1 cell-mediated immune response.

### FUT1 deficiency aggravates corneal opacification and inflammation under desiccating stress

**Fig. 4:**
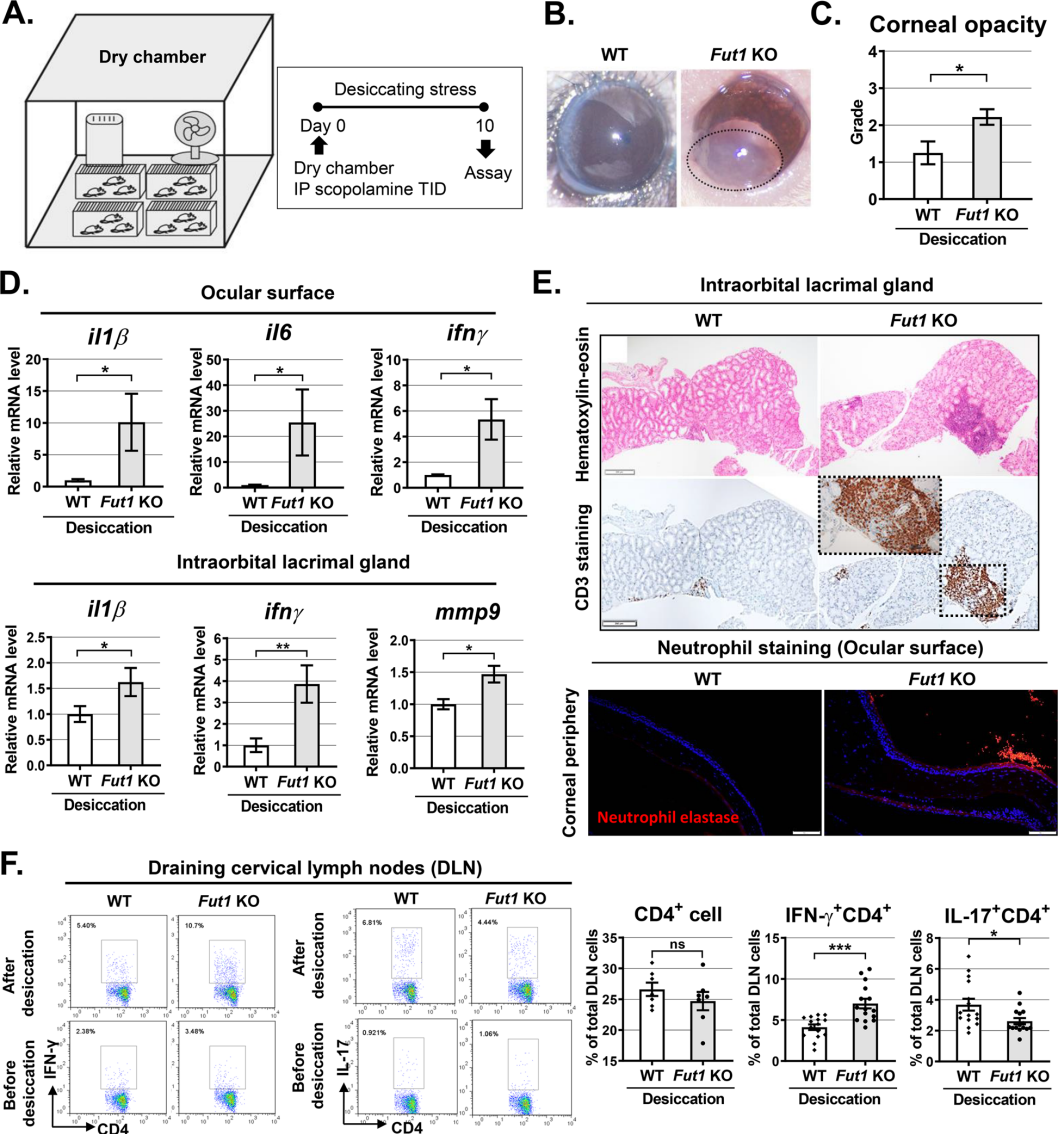
Responses of the ocular surface, lacrimal gland, and DLN to desiccating injury in *Fut1* KO and WT C57BL/6 mice. **a** Illustration of dry chamber with a fan and dehumidifier for induction of desiccation injury and experimental scheme. The 35-week-old mice were placed in a dry chamber for 10 consecutive days with intraperitoneal scopolamine injection three times a day. Ten days after injury initiation, the ocular surface, lacrimal glands, and DLN were assayed. **b** A circle in the photograph depicts the area of corneal opacity observed in *Fut1* KO mice after desiccating injury. **c** Quantification of corneal stromal opacity using the standardized grading system. **d** The transcript levels of inflammatory cytokines in the ocular surface and intraorbital lacrimal gland as determined by real-time RT-PCR. Shown are the relative mRNA levels of each cytokine in *Fut1* KO mice to the levels in WT mice. **e** Hematoxylin-eosin staining and CD3 immunostaining of the intraorbital lacrimal gland after injury (Scale bar, 200 μm). Neutrophil staining of the cornea with anti-neutrophil elastase Ab. Scale bars: 100 μm. **f** Representative and quantitative flow cytometry results for IFN-γ^+^CD4^+^ and IL-17^+^CD4^+^ cells in DLN from *Fut1* KO and WT mice before and after desiccating stress. The percentage of CD4^+^, IFN-γ^+^CD4^+^ or IL-17^+^CD4^+^ cells out of total DLN cells is shown, and a dot represents data from a single individual animal. Bar depicts mean ± SEM. *p < 0.05, **p < 0.01, ***p < 0.001, ns: not significant. Mann–Whitney U test (**c**, **d**) and Student’s *t*-test (**f**) were performed for statistical analysis.

Desiccating stress is one of the most common injuries exerting deleterious effects on the ocular surface and largely mediated by Th1/Th17 effector T-cell responses^20–22^. Since we observed an increased Th1 cell response in *Fut1* KO mice in the above experiments (Fig. 3), we next sought to test whether the ocular surface in FUT1-deficient mice might be more vulnerable to desiccating stress (Fig. 4). To induce desiccating stress to the ocular surface, 35-week-old *Fut1* KO and WT mice were housed in a dry chamber with a forced air flow for 24 h per day and humidity below 35%, and intraperitoneally injected with 0.5 mg scopolamine hydrobromide three times a day for 10 consecutive days (Fig. 4a)^22,23^. *Fut1* deficiency was confirmed in the ocular surface and lacrimal glands of *Fut1* KO mice after desiccating stress (Supplementary Fig. 1). Results showed that desiccating stress-induced corneal epithelial defects and reduced tear production in both *Fut1* KO and WT mice, and the effects were similar between *Fut1* KO and WT mice (Supplementary Fig. 2A). Interestingly, however, corneal opacity was more severe (Fig. 4b, c) and mRNA levels of inflammatory cytokines IL-1β, IL-6, and IFN-γ in the ocular surface were significantly higher in *Fut1* KO mice than in WT controls after desiccating stress (Fig. 4d, Table 1). Similarly, the transcript levels of IL-1β, IFN-γ, and MMP-9 in the intraorbital lacrimal gland were significantly elevated in *Fut1* KO mice (Fig. 4d, Table 1). Consistent with molecular assays, histologic examination demonstrated that CD3^+^ T-cell infiltration in the intraorbital lacrimal gland and neutrophil infiltration adjacent to the corneal epithelium were more prominent in *Fut1* KO mice, compared with WT mice, after desiccating stress (Fig. 4e, Table 1). Similar observation was made with the percentage of IFN-γ^+^CD4^+^ cells in DLNs. The increase of IFN-γ^+^CD4^+^ cells by desiccating stress was more dramatic in *Fut1* KO mice than in WT mice, whereas IL-17^+^CD4^+^ cells were rather decreased in *Fut1* KO mice, leading to an elevated Th1 to Th17 ratio in *Fut1* KO mice after desiccating stress (Fig. 4f, Table 1). The effects of desiccating injury on inflammatory cytokine levels and CD3^+^ T-cell infiltration in the extraorbital lacrimal gland were similar between *Fut1* KO and WT mice (Supplementary Fig. 2B, C, Table 1).

**Table 1:** Alteration of inflammatory responses in *Fut1* knockout (KO) mice compared with wild-type (WT) mice in the steady-state condition (no desiccating stress) and under injury (desiccating stress).

		Fold difference of average values of target expression in *Fut1* KO mice compared with average values in WT (a ratio of *Fut1* KO relative to WT)	
Area	Target	No desiccating stress	Desiccating stress
Ocular surface	IL-1β	2.241	8.847
	IL-6	0.290	25.432
	TNF-α	1.208	1.044
	IFN-γ	3.635	5.338
Extraorbital lacrimal gland	IL-1β	1.127	0.776
	IL-6	0.750	0.946
	TNF-α	2.701	1.090
	IFN-γ	0.920	0.463
	CD3^+^ cell	1.765	0.767
Intraorbital lacrimal gland	IL-1β	1.270	1.562
	IL-6	1.329	1.873
	TNF-α	1.716	1.383
	IFN-γ	1.540	3.860
	CD3^+^ cell	0.728	3.119
Draining lymph node	IFN-γ^+^CD4^+^ cell	1.709	1.671
	IL-17^+^CD4^+^ cell	0.906	0.717

These results collectively suggest that FUT1 deficiency exacerbates Th1 immune response in the eye and DLNs in response to desiccating injury to the ocular surface.

### FUT1 deficiency enhances the activity of splenocytes to stimulate T-cell proliferation

**Fig. 5:**
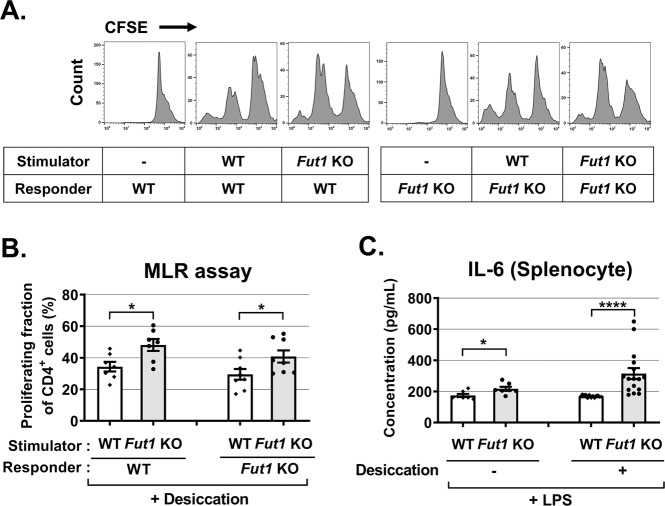
Mixed lymphocyte reaction (MLR) and lipopolysaccharide (LPS) stimulation assays in splenocytes of *Fut1* KO and WT C57BL/6 mice. **a**, **b** Flow cytometric analysis of CFSE dilution after 5 days of mixed cultures of CFSE-labeled CD4^+^ cells (responder) and mitomycin C-pretreated splenocytes (stimulator) from *Fut1* KO or WT mice. **c** The secreted level of IL-6 protein in the cell-free culture supernatant of splenocytes isolated from *Fut1* KO or WT mice after LPS stimulation for 18 h. In some animals, splenocytes were isolated after desiccating stress (as in Fig. 4a). A dot represents the data obtained from an individual animal, and bar depicts mean ± SEM. *p < 0.05, ****p < 0.0001. Student’s *t*-test (**b**) and Mann–Whitney *U* test (**c**) were performed for statistical analysis.

We further examined whether an increased T-cell response in *Fut1* KO mice results either from an increased activity of T cells themselves or from an increased activity of immune cells to stimulate T-cell proliferation. To this end, we performed one-way mixed lymphocyte reaction (MLR) assays (Fig. 5a, b). CD4^+^ cells were purified from the spleen of *Fut1* KO or WT mice that received desiccating injury and used as responder cells after carboxyfluorescein succinimidyl ester (CFSE) labeling. Splenocytes isolated from *Fut1* KO or WT mice after desiccating stress were pretreated with mitomycin C and used as stimulator cells. Assays for CFSE dilution showed that CD4^+^ T-cell proliferation was significantly enhanced upon stimulation with *Fut1* KO splenocytes compared with WT splenocytes regardless of whether CD4^+^ cells were isolated from *Fut1* KO or WT mice (Fig. 5a, b). On the other hand, *Fut1* KO and WT CD4^+^ T cells exhibited the similar proliferating capacity in response to the same stimulator cells (Fig. 5a, b). Therefore, the data indicate that splenocytes from *Fut1* KO mice have higher stimulatory effects on CD4^+^ T cells. In line with this finding, the production of IL-6 was highly elevated in splenocytes from *Fut1* KO mice, compared with the cells from WT mice, in response to lipopolysaccharide (LPS) stimulation (Fig. 5c), which reflects the presence of hyper-stimulatory immune cells in the spleen of *Fut1* KO mice.

## Discussion

Our results demonstrate that *Fut1* gene and its product H2 antigen are constitutively expressed in the ocular surface and lacrimal glands, and FUT1 deficiency leads to the ocular surface inflammation, corneal epithelial defects, and corneal opacification both in steady state and under desiccating stress. Specifically, Th1 immune response was activated in the ocular surface, intraorbital lacrimal gland, and DLNs in FUT1-deficient mice. On the other hand, the extraorbital lacrimal gland, a main gland for aqueous tear production^24^, exhibited normal structure in *Fut1* KO mice and had the similar inflammatory response to WT mice. As such, aqueous tear secretion was not impeded but rather increased in *Fut1* KO mice probably due to reflex tearing in response to corneal epithelial disruption.

The number of conjunctival goblet cells was unchanged in *Fut1* KO mice in our study. Conjunctival goblet cells secrete mucins that are glycoproteins that play an important role in the ocular surface barrier function. Of the secreted mucins, MUC5AC is most prevalently expressed in the ocular surface and tears^25^. A previous study showed that FUT1 overexpression in colon cancer cells catalyzed the addition of α1,2-fucose to MUC5AC, indicating the implication of FUT1 in the glycosylation of MUC5AC^26^. Other studies showed that fucosylation of gastric MUC5AC was lost in *Fut2* KO mice^27^, and FUT2-mediated MUC5AC fucosylation increases mucus viscoelasticity in the airway^28^. Given these roles of FUTs in the mucin glycosylation and functions in various tissues, it is possible to speculate that the function of mucins in the conjunctiva might be impaired by FUT1 deficiency, despite normal number of conjunctival goblet cells in *Fut1* KO mice, and the dysfunction of conjunctival mucins might contribute to the development of corneal epithelial defects in *Fut1* KO mice. Therefore, it would be an interesting subject for further research to investigate mucin glycosylation and function in the conjunctiva in *Fut1* and *Fut2* KO mice.

Glycosylation plays a critical role in the regulation of immune responses through its effects on immune cell adhesion, migration, differentiation, and functional polarization^4^. In this study, we found that IFN-γ^+^CD4^+^ Th1 cells were elevated in ocular DLNs in *Fut1* KO mice under both physiological and pathological conditions, which can be another potential mechanism of corneal epithelial defects and opacity observed in *Fut1* KO mice. Furthermore, *Fut1* KO splenocytes displayed a stronger pro-inflammatory response upon LPS stimulation and had a higher capacity to stimulate CD4^+^ T-cell proliferation in vitro. Thus, the findings support the notion that FUT1 deficiency might activate immune cells in the spleen toward inflammatory phenotypes that provoke T-cell proliferation and favor Th1 cell differentiation. Indeed, it has been shown that terminal and sub-terminal FUTs, including FUT1, are predominantly expressed by M1 inflammatory macrophages, whereas core and O-FUTs are mainly expressed by CD4 T cells^4,10^. Contrary to our findings, however, a study by Li et al. reported that the inhibition of terminal FUT1 and 2 using 2-D-gal (2-Deoxy-D-galactose) precluded M1 macrophage differentiation and repressed its antigen presenting capacity, leading to reduction of Th17 cells and resolution of inflammatory arthritis^10^. By contrast, another study showed that fucosylation inhibition by 2FF (2-deoxy-2-fluoro-L-fucose) upregulated the expression of MHC class II and co-stimulatory molecules on dendritic cells, and 2-D-gal or 2FF treatment activated T cells^4^. Considering these conflicting data, it can be speculated that the process of fucosylation has distinct biological effects depending on tissues and disease contexts.

Apart from its effects on inflammation and immune responses, fucosylation might be associated with epithelial cell migration in the cornea, providing more direct explanation for corneal epithelial disruption observed in *Fut1* KO mice. In support for this possibility, a previous study showed that topical administration of fucose to the ocular surface promoted corneal wound healing^17^. Also, it was reported that H antigens were upregulated in the oral mucosa during wound healing^29^, and small interference RNA of *Fut1* gene inhibited keratinocyte migration in the skin^11^. Moreover, α1,2-fucose in the intestinal epithelium has been shown to be crucial for interactions with luminal microorganisms and creation of a homeostatic microenvironment in the intestine^3,30^. Given the emerging role of fucosylation in the physiology and pathology in the epithelium of the skin, oral mucosa, and intestinal mucosa, future research on the role of FUTs-mediated glycosylation in the ocular epithelium would help understand the homeostatic mechanism and disease pathogenesis in the ocular surface, a frontline barrier for the eye.

In this study, we used *Fut1* KO mice with global abrogation of *Fut1* gene. Therefore, it is difficult to determine whether the corneal epithelial disruption and opacity observed in *Fut1* KO mice might be due to local deficiency of FUT1 in the ocular surface epithelium and stroma or due to FUT1 deficiency in other cell types such as immune cells and nerves. Further experiments using the cell type- or tissue-specific deletion of *Fut1* gene are necessary to confirm the mechanism(s) responsible for the ocular surface phenotype associated with FUT1 deficiency.

## Materials and methods

### Study approval

The experimental protocol was approved by the Institutional Animal Care and Use Committee of Seoul National University (IACUC No. 18-0042).

### Animals

*Fut1* KO mice (B6.129-*Fut1*^tm1Sdo^/J) were purchased from Jackson laboratory (Bar Harbor, ME)^31^. C57BL/6 mice (Jackson laboratory) were used as WT controls. For genetic confirmation of *Fut1* gene knockdown, the genotyping was performed following the protocol from Jackson laboratory.

Both experimental (*Fut1* KO mice) and control groups (C57BL/6 mice) received the same treatment, and no blinding was necessary. The number of animals used in the study was calculated by using G*Power 3.1.9.2 based on our previous experience with a mouse model for dry eye disease and determined to provide 80% statistical power for detection of at least 20% mean difference in tested variables between the two groups.

### Desiccating stress induction

For induction of desiccating stress, 35-week-old *Fut1* KO or C57BL/6 mice were housed in a cage with perforated plastic screen on both sides and dehumidifier for 10 consecutive days. The airflow from an electric fan was allowed into the cage through the screen for 24 h, and humidity was maintained 30–35% inside the cage. In addition, the mice received an intraperitoneal injection of scopolamine hydrobromide (0.5 mg/0.2 mL, Sigma-Aldrich, St. Louis, MO) three times a day for 10 days.

### Clinical examination of corneal epithelial defects and stromal opacity

The ocular surface was observed under an operating microscope and photographed. For corneal epithelial defect quantification, 3% lissamine green vital dye (Lissamine^TM^ Green B dye, Sigma-Aldrich) was applied to the corneal surface, and the stained epithelial defect was graded based on the Oxford scale^32^. Corneal opacity was graded based on the previously-reported scoring system^33^. The grading was carried out by a corneal specialist (K.W.K) in a blind manner.

### Measurement of tear production

Aqueous tear production was quantified by a phenol red thread test. The phenol red–impregnated cotton thread (FCI Ophthalmics, Pembroke, MA) was applied at the lateral canthus for 15 s, and the length of tear-wetted thread was measured.

### Histology

Ocular tissues were stained for H2 antigen with anti-H2 antigen (BRIC231, #sc-59467, Santa Cruz Biotechnology, Dallas, TX), and nuclei were counterstained with DAPI. Fluorescent images were obtained using a microscope (TCS SP8, Leica, Wetzlar, Germany).

The whole eyeball including the forniceal conjunctiva and cornea was excised and fixed in 10% formaldehyde. The tissue was sliced into 4-mm-thick sections and subjected to hematoxylin-eosin, TUNEL, PAS, and neutrophil staining. TUNEL staining was performed using an ApopTag^®^ Plus Fluorescein In Situ Apoptosis Detection Kit (Cat# S7111, EMD Millipore, Burlington, MA) to stain apoptotic cells in the cornea. The number of PAS-stained cells in the conjunctiva was counted in 4 different sections of the forniceal conjunctiva under a microscope (BX53, Olympus, Tokyo, Japan), and the average count per section was determined as the goblet cell count. The counting was performed independently by two researchers (J.S.R and H.J.L) in a blind manner and the average value of the two counts was adopted for analysis. For neutrophil immunostaining, a rat anti-mouse neutrophil elastase antibody (Cat# ab2557, Abcam, Cambridge, MA) and goat anti-rat IgG TRITC (Cat# AP136R, EMD Millipore) were used as primary and secondary antibodies, respectively.

Extraorbital and intraorbital lacrimal glands were excised, fixed in 10% formaldehyde, and embedded in paraffin. Serial 4-μm-thick cross‐sections were subjected to hematoxylin-eosin and CD3 immunostaining (Cat# ab5690, Abcam). The fractional CD3-stained area in Supplementary Fig. 2C was quantified within the outlined lacrimal gland using ImageJ software.

### Real-time RT-PCR

Tissues were lysed in RNA isolation reagent (RNA‐Bee, Tel‐Test Inc., Friendswood, TX) and homogenized with an ultrasound sonicator. Total RNA was extracted with RNeasy Mini kit (Qiagen, Valencia, CA), and an equal amount of RNA was converted to the first‐strand cDNA by reverse transcription (High Capacity RNA-to‐cDNA Kit, Applied Biosystems, Carlsbad, CA). The cDNA was analyzed by real‐time PCR amplification on an ABI 7500 Real-Time PCR System (Applied Biosystems). Mouse‐specific GAPDH was used as an internal control. All probe sets were purchased from Applied Biosystems (TaqMan Gene Expression Assays).

### Western blot

For protein extraction, tissues were lysed with a sonicator in RIPA Buffer (Biosesang, Seongnam, Korea) including a protease inhibitor cocktail (Thermo Fisher Scientific, Waltham, MA). Protein concentration was measured by Bradford assay. 50 µg protein was fractionated by SDS-PAGE on 8-16% Tris-glycine gel (Komabiotech, Seoul, Korea), transferred to methanol-presoaked PVDF membrane (Invitrogen, Waltham, MA), and blotted with antibodies against H2 antigen (1:500, Cat# sc-59467, Santa Cruz Biotechnology), FUT1 (1:500, Cat# sc-21963, Santa Cruz Biotechnology), and β–actin (1:1000, Cat# sc-47778, Santa Cruz Biotechnology).

### Flow cytometry

Single cell suspensions were isolated from tissues and stained with fluorescence-conjugated antibodies against CD4-PE cy7 (Cat# 25-0041, eBioscience, Waltham, MA), IFN‐γ-FITC (Cat# 11-7311, eBioscience), and IL-17-APC (Cat# 17-7177, eBioscience). The stained cells were assayed by a flow cytometer (S1000EXi Flow Cytometer, Stratedigm, San Jose, CA) and analyzed using FlowJo program (Tree Star, Inc., Ashland, OR).

### MLR assay

Splenocytes were isolated from the spleen of mice and used for MLR assays. Responder cells were pre-labeled with 5 μM CFSE (Invitrogen) and stimulator cells pretreated with 25 μg/ml mitomycin C (Sigma-Aldrich). Responder and stimulator cells were mixed at a 1:1 ratio and cultured in RPMI‐1640 medium (WelGENE, Daegu, Korea) containing 10% FBS (Gibco, Grand Island, NY) and 1% penicillin/streptomycin (Sigma-Aldrich) on plates coated with 5 μg/ml anti-CD3/CD28 antibodies (eBioscience) for 5 days. The proliferative response of responder cells was evaluated by measuring CFSE dilution on S1000EXi Flow Cytometer.

### LPS stimulation assay and ELISA

Splenocytes were treated with 1000 ng/mL LPS (InvivoGen, San Diego, CA) and 0.05 mM β-mercaptoethanol for 18 h. The cell-free culture supernatant was assayed for IL-6 concentration by ELISA (IL-6 DuoSet, R&D Systems, Minneapolis, MN).

### Statistical analysis

Prism software v.8.1.2 (GraphPad, La Jolla, CA, USA) was used for statistical tests. To compare the means of two groups, data were analyzed by two-tailed Student’s *t*-test and non-parametric Mann–Whitney *U* test. To compare three or more groups, one-way ANOVA and non-parametric Kruskal–Wallis test were used. Two-way ANOVA test was used to assess differences and interactions between the groups. No animals or samples were excluded from analysis. Data were presented as mean ± SEM. The variance was similar between the groups being statistically compared. Differences were considered significant at *p* < 0.05.

## Supplementary information

Supplementary figure 1

Supplementary figure 2

Supplementary table and figure legends (clean)
