# Supplementary figures and images for: FUT1 deficiency elicits immune dysregulation and corneal opacity in steady state and under stress
Source: Cell Death Dis. 2020 Apr 24;11(4):285. doi: 10.1038/s41419-020-2489-x (PMC7181665; doi:10.1038/s41419-020-2489-x)

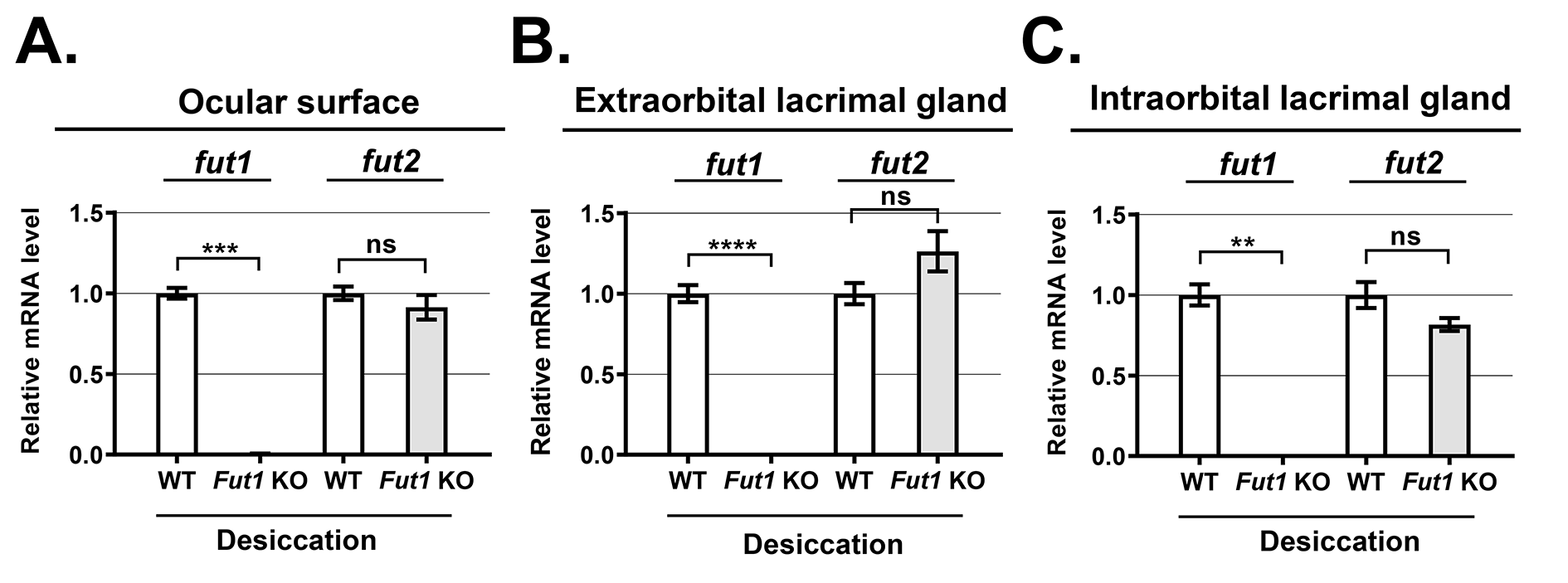

Supplement: Supplementary file 1 — Supplementary figure 1 [file 41419_2020_2489_MOESM1_ESM.tif]

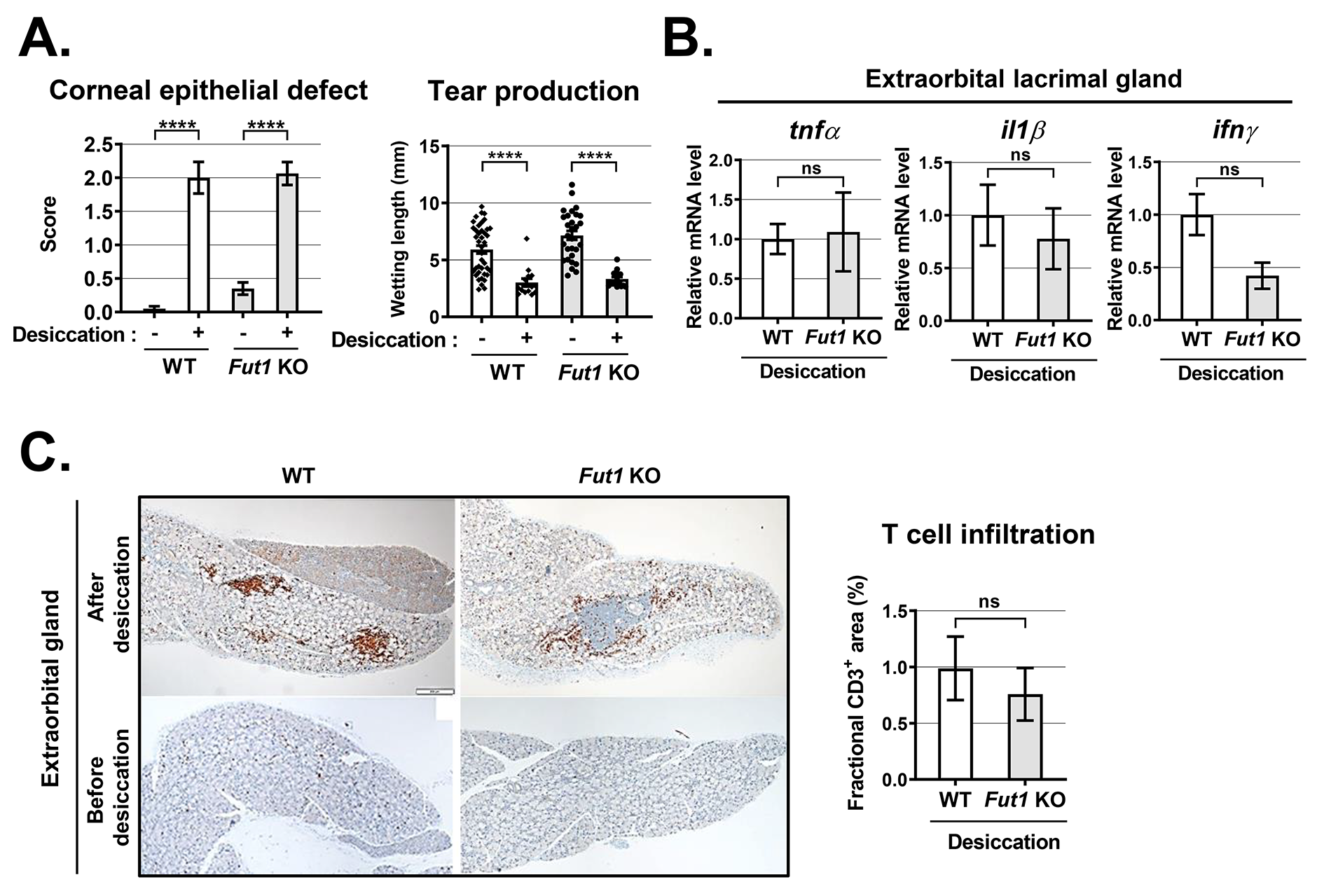

Supplement: Supplementary file 2 — Supplementary figure 2 [file 41419_2020_2489_MOESM2_ESM.tif]
