# Supplementary material for: FUT1 deficiency elicits immune dysregulation and corneal opacity in steady state and under stress
Source: Cell Death Dis. 2020 Apr 24;11(4):285. doi: 10.1038/s41419-020-2489-x (PMC7181665; doi:10.1038/s41419-020-2489-x)
Supplement: Supplementary file 3 — Supplementary table and figure legends (clean) [file 41419_2020_2489_MOESM3_ESM.docx]

**Supplementary table and figure legends**

**Supplementary Table 1.** Two-way analysis of variance (2-way ANOVA) results for the effect of group (*Fut1* KO vs wild-type) and age on corneal epithelial defect, corneal stromal opacity, and tear production.

| Variables | Group (wild-type vs *Fut1* KO mice) | | Age | | Group $\times$ Age | |
| --- | --- | --- | --- | --- | --- | --- |
|  | *F* | *P* | *F* | *P* | *F* | *P* |
| Corneal  epithelial defect | 3.055 | 0.082 | 1.018 | 0.414 | 0.653 | 0.688 |
| Corneal  stromal opacity | 12.004 | <0.001*** | 2.234 | 0.088 | 0.177 | 0.912 |
| Tear production | 0.002 | 0.964 | 3.398 | 0.003** | 1.015 | 0.417 |

***p*<0.01 and ****p*<0.001 with statistical significance.

**Supplementary Figure 1.** Real-time RT PCR analysis for *Fut1 and* *Fut2* genes in the ocular surface (cornea and conjunctiva, **A**), extraorbital (**B**) and intraorbital lacrimal glands (**C**) of 30-week-old *Fut1* KO mice vs wild-type (WT) C57BL/6 mice after desiccating stress. Shown are mRNA levels of each gene in *Fut1* KO mice relative to the levels in WT mice. Data are presented as mean ± SEM. ***p* < 0.01, ****p* < 0.001, *****p* < 0.0001, ns: not significant. Student’s t-test (A, *fut2* in B, C) and Mann-Whitney U test (*fut1* in B, C) were performed for statistical analysis.

**Supplementary Figure 2.** Responses of the ocular surface and extraorbital lacrimal gland to desiccating injury in *Fut1* KO and wild-type (WT) C57BL/6 mice.

**(A)** Corneal epithelial defects as graded after lissamine green vital staining and aqueous tear production as measured by a phenol red thread test in *Fut1* KO and WT mice before and after desiccating stress. A dot depicts data from an individual mouse. **(B)** Real-time RT PCR analysis for mRNA levels of TNF-α, IL-1β, and IFN-γ in the extraorbital lacrimal gland after desiccating stress. Shown are the relative mRNA levels of each cytokine in *Fut1* KO mice to those in WT mice. **(C)** CD3 immunostaining of the extraorbital lacrimal gland before and after desiccating stress. The CD3-stained area in the gland was quantitated as a percentage out of the whole gland area. Data are presented as mean ± SEM. *****p* < 0.0001, ns: not significant. Mann-Whitney U test was performed for statistical analysis.
